# Transcriptomic analysis of the entomopathogenic nematode *Heterorhabditis bacteriophora *TTO1

**DOI:** 10.1186/1471-2164-10-205

**Published:** 2009-04-30

**Authors:** Xiaodong Bai, Byron J Adams, Todd A Ciche, Sandra Clifton, Randy Gaugler, Saskia A Hogenhout, John Spieth, Paul W Sternberg, Richard K Wilson, Parwinder S Grewal

**Affiliations:** 1 Department of Entomology, The Ohio State University – OARDC, Wooster, Ohio, USA; 2 Department of Biology and Evolutionary Ecology Laboratories, Brigham Young University, Provo, UT, USA; 3 Department of Microbiology and Molecular Genetics, Michigan State University, East Lansing, MI, USA; 4 Department of Genetics, Washington University School of Medicine, St Louis, MO, USA; 5 Genome Center, Washington University School of Medicine, St Louis, MO, USA; 6 Department of Entomology, Rutgers University, New Brunswick, NJ, USA; 7 Department of Disease and Stress Biology, The John Innes Centre, Norwich, UK; 8 Howard Hughes Medical Institute and Division of Biology, California Institute of Technology, Pasadena, CA, USA

## Abstract

**Background:**

The entomopathogenic nematode *Heterorhabditis bacteriophora *and its symbiotic bacterium, *Photorhabdus luminescens*, are important biological control agents of insect pests. This nematode-bacterium-insect association represents an emerging tripartite model for research on mutualistic and parasitic symbioses. Elucidation of mechanisms underlying these biological processes may serve as a foundation for improving the biological control potential of the nematode-bacterium complex. This large-scale expressed sequence tag (EST) analysis effort enables gene discovery and development of microsatellite markers. These ESTs will also aid in the annotation of the upcoming complete genome sequence of *H. bacteriophora*.

**Results:**

A total of 31,485 high quality ESTs were generated from cDNA libraries of the adult *H. bacteriophora *TTO1 strain. Cluster analysis revealed the presence of 3,051 contigs and 7,835 singletons, representing 10,886 distinct EST sequences. About 72% of the distinct EST sequences had significant matches (E value < 1e-5) to proteins in GenBank's non-redundant (nr) and Wormpep190 databases. We have identified 12 ESTs corresponding to 8 genes potentially involved in RNA interference, 22 ESTs corresponding to 14 genes potentially involved in dauer-related processes, and 51 ESTs corresponding to 27 genes potentially involved in defense and stress responses. Comparison to ESTs and proteins of free-living nematodes led to the identification of 554 parasitic nematode-specific ESTs in *H. bacteriophora*, among which are those encoding F-box-like/WD-repeat protein theromacin, Bax inhibitor-1-like protein, and PAZ domain containing protein. Gene Ontology terms were assigned to 6,685 of the 10,886 ESTs. A total of 168 microsatellite loci were identified with primers designable for 141 loci.

**Conclusion:**

A total of 10,886 distinct EST sequences were identified from adult *H. bacteriophora *cDNA libraries. BLAST searches revealed ESTs potentially involved in parasitism, RNA interference, defense responses, stress responses, and dauer-related processes. The putative microsatellite markers identified in *H. bacteriophora *ESTs will enable genetic mapping and population genetic studies. These genomic resources provide the material base necessary for genome annotation, microarray development, and in-depth gene functional analysis.

## Background

The entomopathogenic nematode, *Heterorhabditis bacteriophora*, and its mutualistic bacterium, *Photorhabdus luminescens*, are important biological control agents of insect pests [1] and represent an emerging model for research on mutualistic and parasitic symbiosis [2,3]. The use of *H. bacteriophora *as a biological control agent is hampered by its susceptibility to environmental extremes including temperature, desiccation, and UV radiation, differences in virulence towards different insect pests, and short shelf life. Elucidation of molecular mechanisms underlying these biological processes may serve as a foundation for improving the biological control potential of the nematode-bacterium complex.

The entomopathogenic nematode *H. bacteriophora *has a distinct life style. The infective juveniles (IJs) or dauer juveniles (DJs) persist in soil in search of a suitable insect host [4]. Following entry into the insect host through natural body openings and cuticle, the IJs regurgitate the symbiotic bacteria into the insect hemocoel [5]. The bacteria kill the insect host via septicemia, usually within 24–48 h [5]. The nematodes feed on the multiplying bacteria and disintegrated host tissues and produce 1 to 3 generations within the cadaver. When the food source depletes and nematode density reaches a threshold, next-generation IJs are formed which exit the cadaver in search of a new host [3].

In contrast to the closely related genetic model *Caenorhabditis elegans*, few genomic resources are available for *H. bacteriophora*. However, some progress has been made over the past few years with the generation and analysis of ~1,000 ESTs from *H. bacteriophora *GPS11 strain [3,6], the start of an *H. bacteriophora *complete genome sequence project (supported by the National Human Genome Research Institute), and the development of a reverse genetics tool using RNA interference [7]. Release of the genomes of *C. elegans *[8], *C. briggsae *[9], *Brugia malayi *[10], bacterium *Photorhabdus luminescens *subsp. *laumondii *TTO1 (obligate endosymbiont) [11] and over 1 million ESTs from various nematode species deposited in GenBank offers unprecedented opportunities for the genetics of entomopathogenic nematodes. Here, we report on the construction of cDNA libraries from *H. bacteriophora *TTO1 adult hermaphrodites and the generation and analysis of 31,485 ESTs. The TTO1 strain is different from GPS11 strain in insect toxicity and their symbiotic bacteria (Grewal et al., unpublished). ESTs are valuable for gene discovery and can also be used in the identification of microsatellite markers [12]. Therefore, we also identified microsatellite loci in *H. bacteriophora *ESTs for use in population genetic studies. In addition, ESTs will also aid the prediction of protein-coding genes in the annotation of complete genomes. However, domain identification, secretome prediction, phylogenetic and evolution analysis on the ESTs that are of short-length and low coverage are not very informative, and therefore were not performed.

## Results

### Generation of ESTs and assembly

A total of 31,586 ESTs from adult *H. bacteriophora *TTO1-M13e strain were generated from cDNA libraries and were deposited in GenBank under the accession numbers [GenBank:EG025323] – [GenBank:EG025806], [GenBank:ES408468] – [GenBank:ES414355], [GenBank:ES738967] – [GenBank:ES744677], [GenBank:EX006911] – [GenBank:EX015306], [GenBank:EX910019] – [GenBank:EX916843], and [GenBank:FF678120] – [GenBank:FF681586]. The removal of vector and short length (20 bp or less) sequences resulted in 31,485 high-quality ESTs with an average length of 531 bp. The cumulative length of all high-quality EST sequences was 16,713,919 bases.

**Figure 1:**
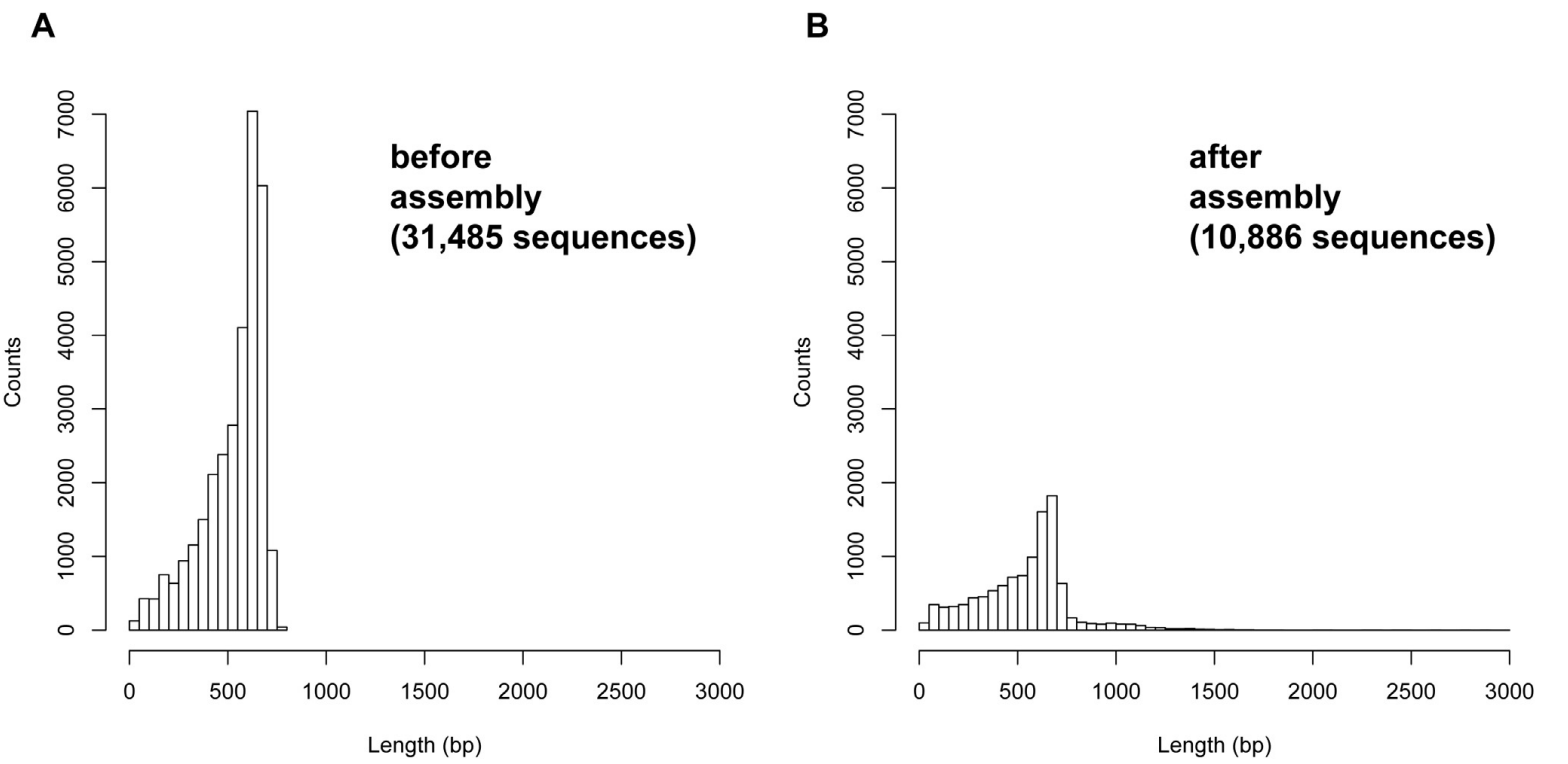
**Length distribution of *H. bacteriophora *ESTs before and after assembly**.

The ESTs were subjected to cluster analysis using Vector NTI Advance™ 10 (Invitrogen), with the final assembly generated by Vector NTI Advance™ 10. The final assembly contained 3,051 contigs generated from 23,650 ESTs and 7,835 singletons (Table 1). The contigs consisted of 2 to 283 ESTs (See additional file 1: Components of *H. bacteriophora *assembled contigs) with lengths ranging from 60 to 2,856 bp and a mean of 743 bp (Table 1). The average length of singletons was 461 bp (Figure 1). In total, we identified 10,886 distinct EST sequences.

**Table 1:** Summary statistics of ESTs generated from *H. bacteriophora*

Feature	Value
Number of high quality ESTs	31,485
Average length of high quality ESTs (bp)	531
Number of contigs	3,051
Number of ESTs in contigs	23,650
Number of singletons	7,835
Number of distinct sequences	10,886
Average length of contigs (bp)	743
Average length of singletons (bp)	461

### Putative functional identifications of the ESTs

**Figure 2:**
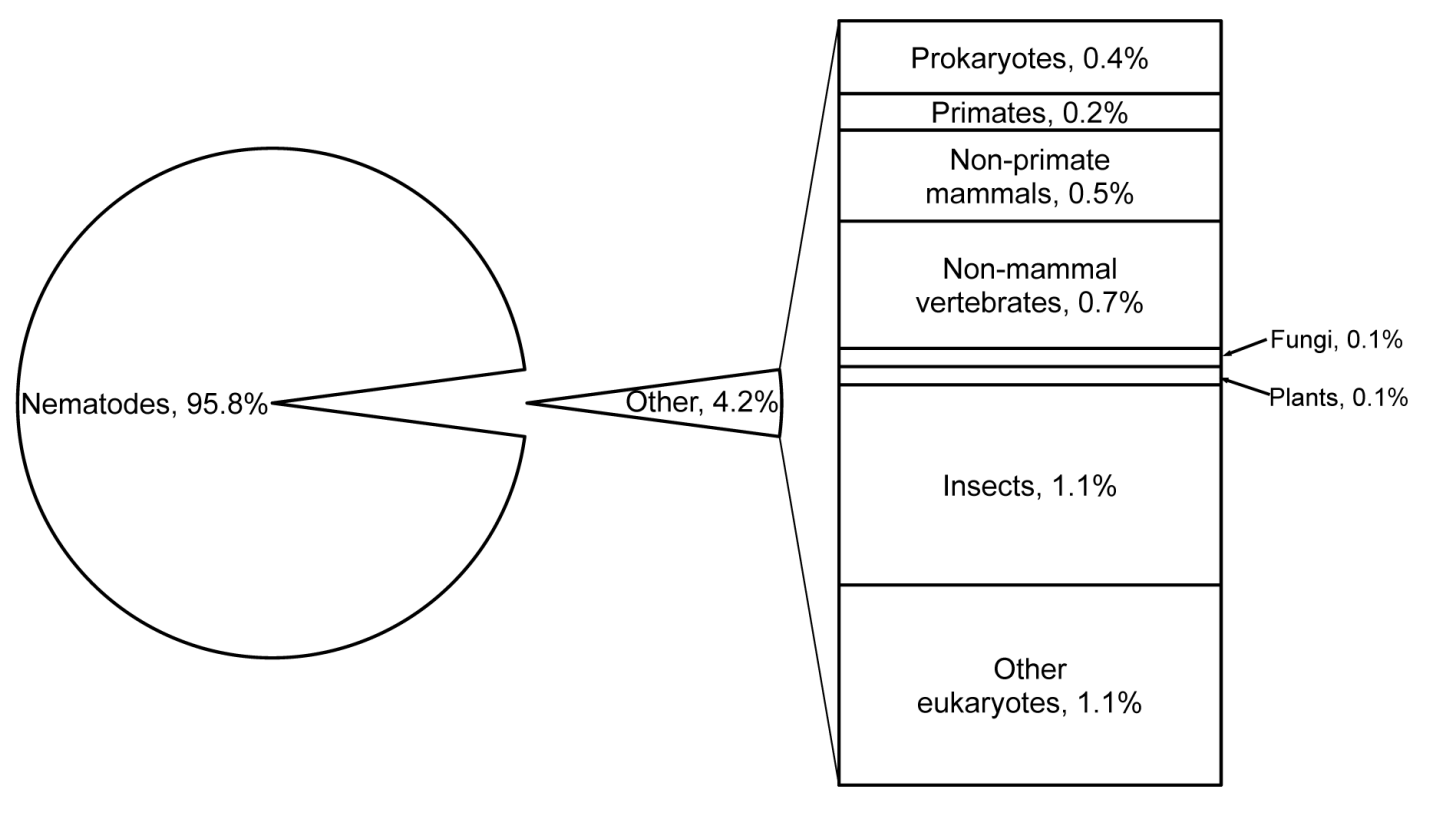
**Categories of organisms with significant protein matches of *H. bacteriophora *distinct ESTs**. The percentage was calculated considering the total number of *H. bacteriophora *distinct ESTs having significant matches (E value < 1e-5) as 100%.

In order to assess the putative identities, all distinct ESTs were subjected to BLASTx sequence similarity searches against GenBank's nr database and Wormpep190 database consisting of extensively curated *C. elegans *proteins from Wormbase [13]. Of the 10,886 distinct ESTs, 7,828 (71.9%) had significant matches (E value cutoff 1e-5) to proteins in GenBank's nr database. As expected, most of the best matches (95.9%) were to nematode proteins (Figure 2). A small proportion (0.3%) of the best matches was to prokaryotic proteins with localized sequence similarity ranging from 28% to 98% and a median of 81% (Table 2) and the remaining 3.8% of the best matches was to other eukaryotes including humans, fungi, plants, and insects. Of the remaining 3,058 *H. bacteriophora *distinct ESTs, 119 had significant matches to nucleotide sequences in GenBank nt database, including 31 that matched to mitochondrial genes.

**Table 2:** Summary of *H. bacteriophora *distinctive ESTs matching to prokaryotic proteins

	Best BLASTx match			
	
*H. bacteriophora *distinctive ESTs	Description	Organism	E value	Identities^a^
Contig131	MTA/SAH nucleosidase	*Enterococcus faecalis *V583	2e-44	308/507, 86%
ES410513	MTA/SAH nucleosidase	*Enterococcus faecalis *V583	6e-11	125/227, 86%
ES410515	MTA/SAH nucleosidase	*Enterococcus faecalis *V583	7e-18	227/231, 67%
ES410516	MTA/SAH nucleosidase	*Enterococcus faecalis *V583	2e-16	161/171, 81%
ES410519	MTA/SAH nucleosidase	*Enterococcus faecalis *V583	8e-17	158/251, 87%
ES410521	MTA/SAH nucleosidase	*Enterococcus faecalis *V583	2e-25	188/288, 95%
ES410525	MTA/SAH nucleosidase	*Enterococcus faecalis *V583	9e-15	149/249, 84%
Contig145	DpnD protein	*Streptococcus pneumoniae *TIGR4	7e-21	152/375, 98%
Contig672	Adenylate cyclase	*Burkholderia oklahomensis *EO147	2e-16	500/962, 33%
Contig1258	GTP-binding protein HSR1-related	*Thauera *sp. MZ1T	4e-21	596/649, 31%
Contig1517	hypothetical protein Bmal10_03004924	*Burkholderia mallei *10399	2e-10	230/809, 47%
Contig2004	binding-protein-dependent transport systems inner membrane component	*Pyrobaculum calidifontis *JCM 11548	2e-15	290/325, 39%
Contig2104	conserved hypothetical protein	uncultured beta proteobacterium CBNPD1 BAC clone 578	2e-25	185/390, 85%
EG025466	Hypothetical protein COLAER_01671	*Collinsella aerofaciens *ATCC 25986	2e-41	404/436, 64%
ES410527	post-segregation antitoxin CcdA	*Escherichia coli *E24377A	2e-21	137/325, 91%
ES410742	hypothetical protein MS53_0454	*Mycoplasma synoviae *53	9e-06	539/686, 28%
ES410920	Sodium/pantothenate symporter	*Rickettsia akari *str. Hartford	0	44/278, 60%
ES739471	conserved hypothetical protein	*Burkholderia ambifaria *MC40-6	0	44/628, 60%
EX911257	transposase, IS4 family protein	*Escherichia coli *O157:H7 str. EC869	4e-52	311/657, 98%
FF681770	rod shape-determining protein	*Xanthomonas oryzae *pv. oryzae KACC10331	2e-26	272/274, 76%
FF681812	ATP-dependent Clp protease proteolytic subunit	*Rhodococcus *sp. RHA1	3e-12	116/168, 82%
FF681935	Ribonucleotide reductase, alpha subunit	*Leuconostoc citreum *KM20	8e-11	155/175, 66%
FF681952	rod shape-determining protein	*Xanthomonas oryzae *pv. oryzae KACC10331	4e-12	119/191, 85%
FF681963	UDP-N-acetylglucosamine 1-carboxyvinyltransferase	*Tenacibaculum *sp. MED152	3e-15	179/183, 65%
FF682197	UDP-N-acetylglucosamine 1-carboxyvinyltransferase	*Algoriphagus *sp. PR1	3e-17	179/221, 70%
FF682207	rod shape-determining protein	*Xylella fastidiosa *9a5c	1e-26	248/269, 78%

a. "Identities" was presented as "length of aligned *H. bacteriophora *EST"/"full length of *H. bacteriophora *EST", percentage of identities over the aligned sequences.

**Figure 3:**
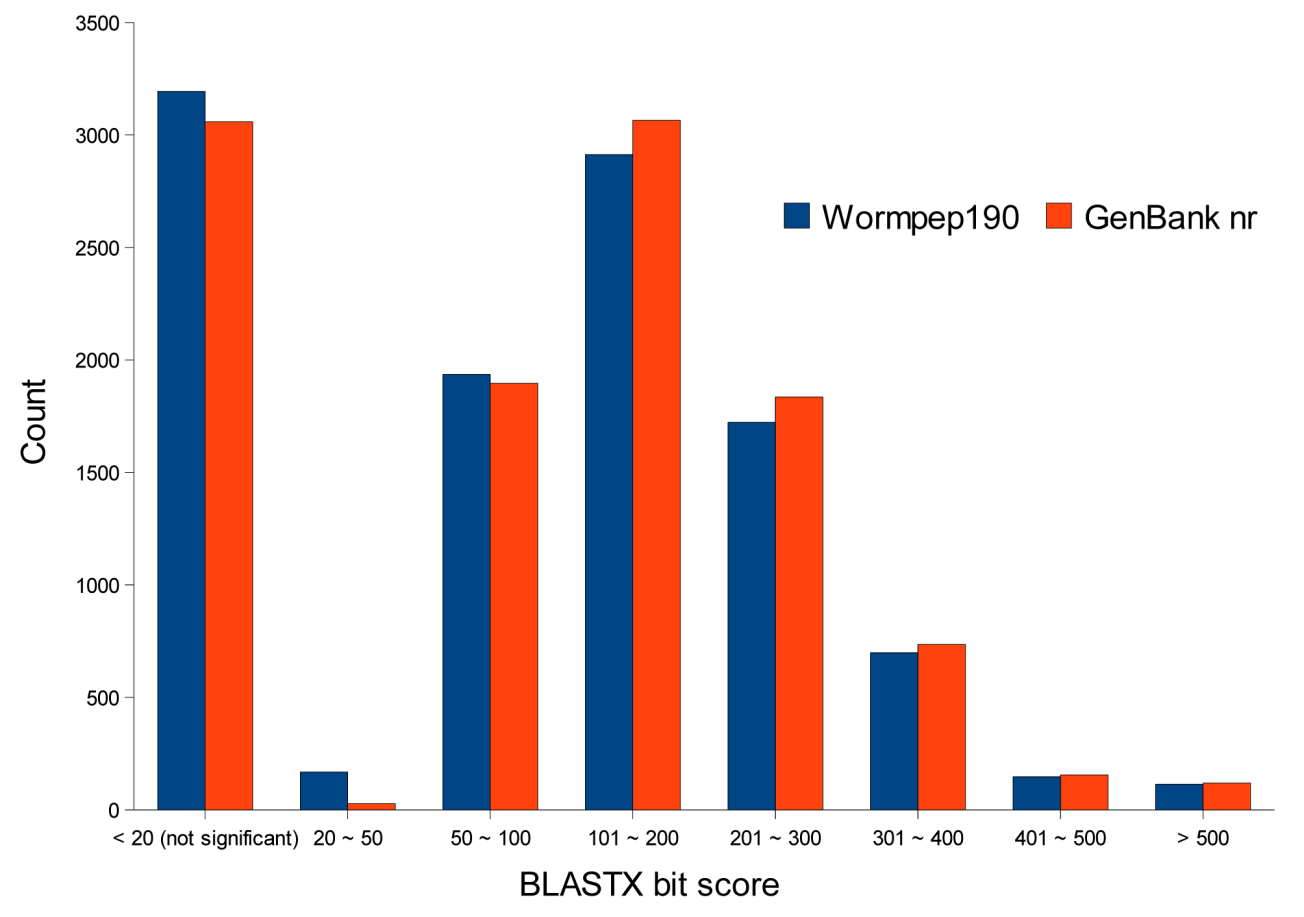
**Distribution of bit scores from the best matches of BLASTx searches of *H. bacteriophora *distinct ESTs**. BLASTx searches with bit scores less than 20 were not significant, roughly corresponding to E value > 1e-5.

**Figure 4:**
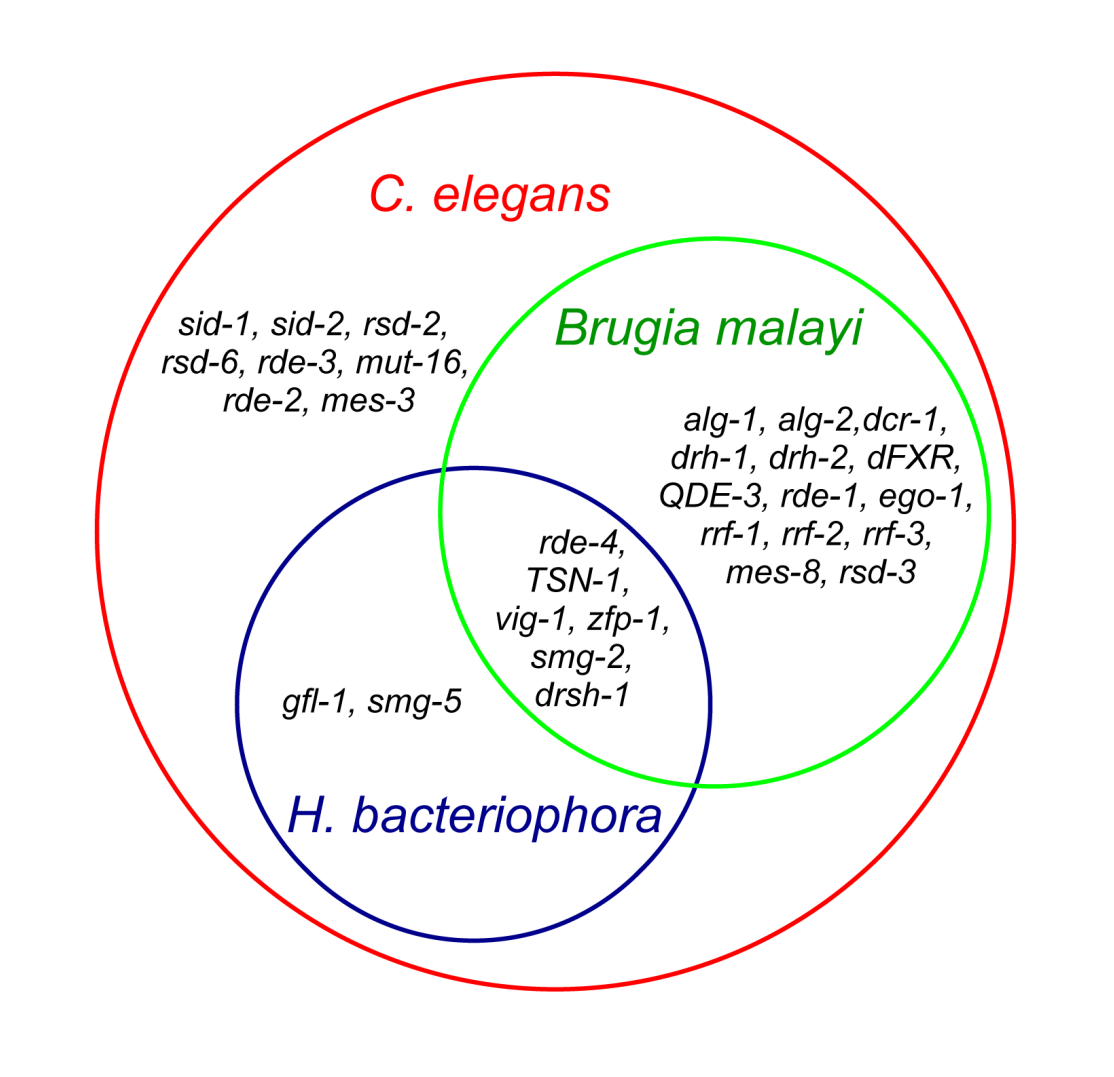
**RNA interference related genes identified in *H. bacteriophora *distinct ESTs**. Genes in red circle were identified in *C. elegans*. Genes in green circle were identified in *B. malayi*. Genes in blue circle were identified in *H. bacteriophora*.

The similarity search against *C. elegans*-specific database Wormpep190 returned essentially similar results (Figure 3). Of the 10,886 *H. bacteriophora *distinct ESTs, 7,699 (70.7%) had significant matches (E value cutoff 1e-5) to 4,460 *C. elegans *proteins in Wormpep190 database. Based on sequence similarity results, 12 *H. bacteriophora *ESTs were identified to be involved in RNA interference (RNAi) pathway (Table 3). The currently identified *H. bacteriophora *RNAi genes are a small portion of those identified in *C. elegans *and *B. malayi *(Figure 4). We also identified 22 ESTs corresponding to 14 genes potentially involved in dauer-related processes (Table 4) and 51 ESTs corresponding to 27 genes involved in defense and stress responses (see Additional File 2: *H. bacteriophora *distinct ESTs similar to *C. elegans *genes involved in defense and stress responses).

**Table 3:** *H. bacteriophora *distinct ESTs similar to *C. elegans *genes involved in RNA interference

*H. bacteriophora *distinct EST	*C. elegans *homolog	Putative function	E value	Similarity^a^
FF679415	WBGene00009163 (*drsh-1*)	Predicted RNase III-type ribonuclease that is orthologous to *Drosophila *and human Drosha	5e-12	555/628, 43%
ES739421	WBGene00001585 (*gfl-1*)	Ortholog of human GAS41.	1e-73	591/599, 77%
ES741260	WBGene00004326 (*rde-4*)	dsRNA binding protein (dsRBP)	7e-08	369/505, 50%
EX913423	WBGene00004880 (*smg-2*)	RNA helicase nonsense mRNA reducing factor	2e-47	509/576, 66%
Contig658	WBGene00004883 (*smg-5*)	Component of nonsense-mediated mRNA decay (NMD) pathway	5e-22	498/672, 61%
EX014403	WBGene00006626 (*TSN-1*)	Component of the RNA-induced silencing complex (RISC)	2e-64	651/652, 71%
Contig211	WBGene00006924 (*vig-1*)	Predicted RNA-binding protein orthologous to *Drosophila *VIG (Vasa Intronic Gene)	2e-26	594/1573, 45%
EX915778	WBGene00006975 (*zfp-1*)	A leucine zipper, zinc finger, and PHD/LAP domain-containing protein	1e-10	144/567, 83%
EX912737	WBGene00006975 (*zfp-1*)	A leucine zipper, zinc finger, and PHD/LAP domain-containing protein	5e-09	594/607, 38%

a. "Similarity" was presented as "length of aligned *H. bacteriophora *EST"/"full length of *H. bacteriophora *EST", percentage of positives over the aligned sequences.

**Table 4:** *H. bacteriophora *distinct ESTs similar to *C. elegans *genes involved in dauer larval development, dauer entry, and dauer exit

*H. bacteriophora *distinct EST	Matching *C. elegans *gene	Description	BLASTx E value	Alignment^a^	% similarity
**Dauer larval development**					
Contig58	WBGene00018294	*atgr-18 *WD40 repeat-containing protein	6e-70	(55–624)/625; (16–208)/394	87%
Contig349	WBGene00010882	*atgr-7 *E1 ubiquitin-activating-like enzyme	4e-85	(3–743)/743; (373–609)/647	77%
EX010274	WBGene00010882	*atgr-7 *E1 ubiquitin-activating-like enzyme	4e-44	(31–597)/601; (2–197)/647	67%
Contig1114	WBGene00000247	*bec-1 *beclin (human autophagy) homolog	7e-33	(214-747)/748; (2–181)/375	62%
EX916779	WBGene00000247	*bec-1 *beclin (human autophagy) homolog	6e-10	(215–442)/444; (2–79)/375	59%
Contig1259	WBGene00004860	*sma-6 *Activin receptor	5e-15	(72–518)/693; (505–634)/636	54%
Contig1603	WBGene00006786	*unc-51 *serine/threonine protein kinase	1e-122	(222–932)/1459; (39–275)/856	81%
ES740745	WBGene00006786	*unc-51 *serine/threonine protein kinase	2e-35	(101–460)/552; (1–120)/856	68%
FF681332	WBGene00006786	*unc-51 *serine/threonine protein kinase	4e-63	(41–502)/503; (671–833)/856	83%
Contig2206	WBGene00004736	*sca-1 *E1–E2 ATPases	3e-77	(2–460)/608; (844–996)/1004	95%
EX012478	WBGene00004736	*sca-1 *E1–E2 ATPases	5e-89	(37–597)/599; (1–187)/1004	94%
FF679293	WBGene00004736	*sca-1 *E1–E2 ATPases	1e-50	(1–399)/430; (780–912)/1004	81%
ES739311	WBGene00006600	*tph-1 *tryptophan hydroxylase	2e-63	(12–587)/587; (23–217)/532	81%
ES741003	WBGene00000090	*age-1 *phosphatidylinositol 3-kinase	3e-09	(30–128)/497; (1113–1145)/1146	85%
EX909983	WBGene00000903	*daf-7 *transforming growth factor beta proteins	1e-39	(9–491)/643; (190–350)/350	65%
FF678376	WBGene00006527	*tax-6 *calcineurin A	3e-56	(2–334)/348; (358–468)/597	95%
FF681873	WBGene00003965	*pdk-1 *3-phosphoinositide-dependent kinase 1	5e-22	(9–221)/244; (517–587)/636	79%
**Dauer entry**					
Contig710	WBGene00004789	*sgk-1 *serine/threonine protein kinase	1e-103	(1–720)/1010; (220–459)/463	84%
EX913794	WBGene00004789	*sgk-1 *serine/threonine protein kinase	6e-68	(20–664)/664; (17–231)/463	74%
ES739212	WBGene00000102	*akt-1 *serine/threonine kinase	5e-60	(128–526)/630; (1–132)/546	89%
**Dauer exit**					
Contig933	WBGene00003831	*nuo-1 *NADH-ubiquinone oxidoreductase	1e-108	(9–659)/676; (1–219)/479	90%
EX007842	WBGene00003831	*nuo-1 *NADH-ubiquinone oxidoreductase	7e-87	(3–488)/645; (317–478)/479	97%

a. The alignment is presented in the following format: (*H. bacteriophora *EST start coordinate – *H. bacteriophora *EST end coordinate)/*H. bacteriophora *length; (*C. elegans *start coordinate – *C. elegans *end coordinate)/*C. elegans *length.

### Identification of parasitic nematode-specific ESTs

**Figure 5:**
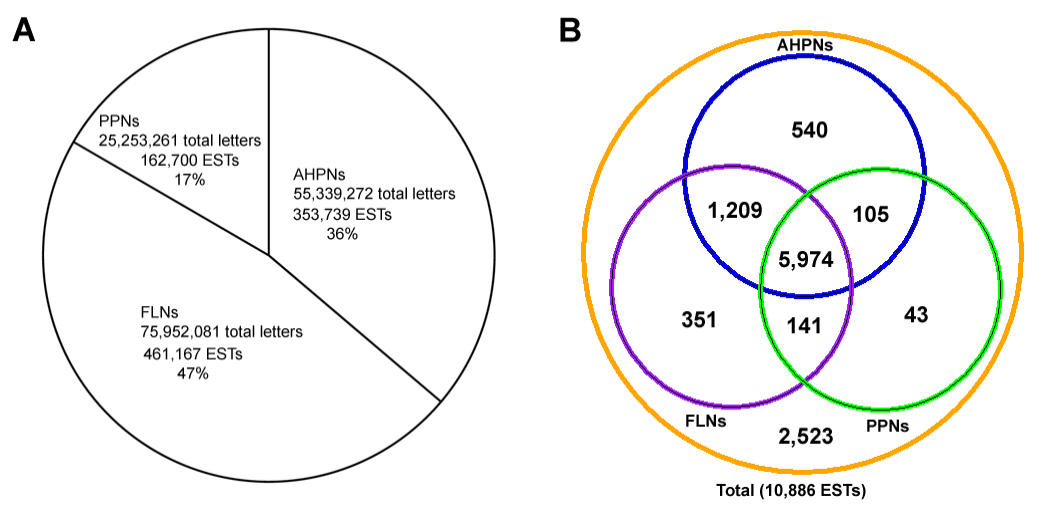
**Summary of comparison between *H. bacteriophora *distinct ESTs and other ESTs from other nematode groups**. (A) Summary of EST databases build from publicly-available animal- and human-parasitic nematodes (AHPNs), plant-parasitic nematodes (PPNs), and free-living nematodes (FLNs). (B) Results of EST comparison with numbers of matches to the ESTs of different categories. Numbers in brown circle are the total numbers of *H. bacteriophora *distinct ESTs. Numbers in green circle are the numbers of *H. bacteriophora *distinct ESTs matching to ESTs from PPNs. Numbers in purple circle are the numbers of *H. bacteriophora *ESTs matching to ESTs from FLNs. Numbers in blue circle are the numbers of *H. bacteriophora *ESTs matching to ESTs from AHPNs. All matches were tBLASTx hits better than E value of 1e-5.

In order to identify parasitic nematode-specific ESTs, a comparison of *H. bacteriophora *ESTs to all nematode EST sequences from GenBank (see additional file 3: Nematode species that ESTs used in the analysis came from) was performed. The nematode taxa having ESTs were divided into animal- and human-parasitic nematodes (AHPNs), plant-parasitic nematodes (PPNs), and free-living nematodes (FLNs) to enable a more informative comparison using tBLASTx algorithm with an E value cutoff of 1e-5 (Figure 5A). Of the 10,886 *H. bacteriophora *ESTs, 2,523 had no matches to nematode ESTs (Figure 5B) of which 2,371 had no matches to proteins but 152 had matches to proteins of other organisms. There were 351 *H. bacteriophora *ESTs matching to ESTs of FLNs only, which encoded proteins potentially involved in processes shared by FLNs and entomopathogenic nematodes, such as dauer formation and response to environmental stresses. There were 540 *H. bacteriophora *ESTs matching only to ESTs of AHPNs, 43 matching only to ESTs of PPNs, and 105 matching only to ESTs of AHPNs and PPNs. Therefore, there were collectively 688 *H. bacteriophora *ESTs not matching to any of the ESTs of FLNs. When these 688 *H. bacteriophora *ESTs were searched against GenBank's nr database using BLASTx algorithm we found that 554 had no matches to proteins of FLNs. These 554 *H. bacteriophora *ESTs were designated as parasitic nematode-specific ESTs and listed in the additional file 4: Summary of BLASTx identification of parasitic nematode-specific *H. bacteriophora *ESTs. Among these 554 ESTs, 476 (86%) matched to ESTs from clade V parasitic nematodes and the remaining 78 ESTs (14%) matched to ESTs from parasitic nematodes in other clades. Among these parasitic nematode-specific ESTs, 142 had matches to proteins of other organisms, enabling putative function assignment. Among these ESTs are those encoding F-box-like/WD-repeat protein, theromacin, Bax inhibitor-1-like protein, and PAZ domain containing protein, which represent interesting targets for in-depth functional analysis. The remaining 412 had no matches to any protein in the current databases, and are thus considered novel sequences.

### Gene ontology annotation

Gene Ontology (GO) terms were assigned to 6,685 distinct ESTs with BLASTx search against the April 2008 release of GO database (see additional file 5: Summary of GO assignment of *H. bacteriophora *distinct ESTs). The GO assignment included 1,117 Biological Process terms assigned 13,438 times to 4,653 distinct ESTs, 244 Cellular Component terms assigned 2,454 times to 1,778 distinct ESTs, and 669 Molecular Function terms assigned 4,035 times to 3,190 distinct ESTs. "Embryonic development ending in birth or egg hatching" (40.9%) was the most dominant term out of the 4,653 distinct ESTs assigned to Biological Process GO category, followed by "nematode larval development" (28.3%), "positive regulation of growth rate" (27.6%), "reproduction" (24.7%), and "growth" (23,6%). Biological Process term associations of *H. bacteriophora *distinct ESTs that may present potential interests included: (i) 164 with "determination of adult life span"; (ii) 22 with "defense response"; (iii) 22 with dauer-related biological processes, including "dauer larval development", "dauer entry", and "dauer exit"; and (iv) 37 with stress-related biological processes. Protein binding (53.8%) was the most dominant term among the 3,190 *H. bacteriophora *ESTs annotated to the Molecular Function category, followed by identical protein binding (2.6%) and cytochrome-c oxidase activity (2.5%). Among the 1,778 *H. bacteriophora *ESTs annotated to Cellular Component category, 221 were nuclear and 218 were in cytoplasm. The GO assignment for each *H. bacteriophora *EST is given in additional file 4: Summary of GO assignment of *H. bacteriophora *distinct ESTs.

### Microsatellite-containing ESTs

In total, we identified 168 microsatellite loci from 157 *H. bacteriophora *distinct EST sequences. Among these 157 *H. bacteriophora *ESTs, 77 had no matches to proteins in GenBank's nr database. The identified microsatellites were di-nucleotide (39.4%), tri-nucleotide (46.3%), tetra-nucleotide (2.7%), penta-nucleotide (0.5%), and hexa-nucleotide (0.5%) (Table 5). Among the 168 microsatellite loci, 141 had good flanking sequences for primer design while the remaining 27 had either short flanking sequences or the flanking sequences had too low GC contents for primer design (see additional file 6: Summary of microsatellite loci identified in *H. bacteriophora *distinct ESTs). The primers designed for the 141 microsatellite loci are potentially useful for genetic linkage mapping and population genetic studies.

**Table 5:** Summary of microsatellite loci identified from *H. bacteriophora *distinct ESTs

Number of repeats^a^	Di-nt unit	Tri-nt unit	Tetra-nt unit	Penta-nt unit	Hexa-nt unit
5	0	55	4	1	0
6	29	11	1	0	0
7	16	11	0	0	0
8	10	6	0	0	0
9	7	1	0	0	1
10	2	2	0	0	0
11	6	0	0	0	0
12	1	0	0	0	0
14	1	0	0	0	0
15	0	1	0	0	0
16	1	0	0	0	0
: :					
31	1	0	0	0	0

Sub-total	74	87	5	1	1
Percentage	39.4%	46.3%	2.7%	0.5%	0.5%

a. The numbers omitted from this column indicate that there were no microsatellite loci containing such repeats.

## Discussion

This work produced a total of 31,485 high quality ESTs representing 10,886 distinct sequences. Sequence similarity searches of *H. bacteriophora *distinct ESTs showed 71.9% (7,828) matches to proteins from GenBank's nr database. The remaining 28.1% *H. bacteriophora *distinct ESTs represented novel genes yet to be assigned a function, demonstrating enormous novel gene discovery potential of this EST study. Among *H. bacteriophora *distinct ESTs having matches to proteins of other organisms in GenBank's nr database, a vast majority (95.9%) matched nematode proteins. About 71% (7,699) *H. bacteriophora *distinct ESTs match to 4,460 proteins from Wormpep190 that contains 23,771 extensively curated *C. elegans *proteins. *H. bacteriophora *homologs in *C. elegans *represent 18.8% of proteins in *C. elegans*. This finding suggests that *H. bacteriophora *and *C. elegans *have vastly different in proteomes, which may be explained in part by free-living versus parasitic life styles.

Interestingly, 26 distinct ESTs (0.3%) matched to proteins from various prokaryotic organisms (Table 2), all of which had less than 100% local sequence identities to prokaryotic sequences. These transcripts could result from horizontal gene transfer from bacteria encountered by *H. bacteriophora *during its life cycle. None of these ESTs matched to genes or proteins of *P. luminescens *subsp. *laumondii *TTO1, the natural symbiont of *H. bacteriophora *TTO1 [11]. Given the fact that poly(A) RNA was used in EST sequencing and the prokaryotic sequences were less than 100% identical to known prokaryotes, the possibility that these sequences are contaminants from other bacteria is low, although the possibility cannot be ruled out completely. The identification of sequences of putative prokaryotic origin in *H. bacteriophora *ESTs are consistent with our previous observations [6] and those observed in plant parasitic nematodes [14]. The putative prokaryotic origin of these sequences could be tested more rigorously once the complete genome sequence becomes available.

Sequence similarity searches of *H. bacteriophora *distinct ESTs against ESTs of other nematodes and proteins revealed the presence of 554 parasitic nematode-specific ESTs (Additional file 3). Eighty-six percent of these ESTs matched ESTs from parasitic nematodes in clade V. Taken into consideration the fact that most FLNs included in this study were also from clade V, we are confident that these ESTs reflect the differences between parasitic and free-living nematodes and are not the result of phylogenetic constraint. These 554 *H. bacteriophora *ESTs had sequence similarities with ESTs from other parasitic nematodes, suggesting that these genes may participate in parasitism-related activities. Although the 2,523 ESTs without matches to any nematode ESTs could be *H. bacteriophora *specific, we hesitate to consider them as parasitic nematode specific at this time because they lack sequence similarity to ESTs of parasitic nematodes. Among these, the 2,371 ESTs without matches to any proteins in the current GenBank nr database represent potentially novel *H. bacteriophora *genes, and 81 of these were identified in the EST dataset of *H. bacteriophora *GPS11 strain [3,6]. These findings suggest the enormous potential of discovering new genes and gene functions, genetic networks, and metabolic pathways specific to *H. bacteriophora *and other entomopathogenic nematodes. The identification of *H. bacteriophora *ESTs shared with other parasitic nematodes through our EST comparison opens the door for conducting in-depth research on gene functions that will ultimately elucidate the parasitic nematode-specific biological processes.

Among the 554 parasitic nematode-specific ESTs are those encoding F-box-like/WD-repeat protein, theromacin, Bax inhibitor-1-like protein, and PAZ domain containing protein. EST FF678238 encodes a homolog of the F-box-like/ED-repeat protein in *Brugia malayi *[10]. The WD-repeat is commonly associated with F-box domain that mediates protein-protein interactions in a variety of contexts such as polyubiquitination [15]. EST FF678397 encodes a protein similar to the PAZ domain containing protein in *Brugia malayi *[10]. The PAZ (Piwi, Argonaut and Zwille) domain has nucleic acid-binding capability and is potentially involved in post-transcriptional gene silencing [16]. Further investigation is needed to elucidate the functions of these two ESTs with common domains and whether they are related to parasitic nematode-specific processes.

Two other parasitic nematode-specific ESTs are Contig2528 and Contig1066. Contig2528 encodes a homolog of theromacin in the segmented worm *Theromyzon tessulatum *[17]. Theromacin is a novel antimicrobial peptide acting against Gram-positive bacteria but without any similarities to other known antimicrobial peptides [17]. *H. bacteriophora *TTO1 is obligately symbiotic with *Photorhabdus luminescens *subsp. *laumondii *TTO1 in natural environments. The production of an antimicrobial peptide could help establish the symbiotic relationship by selectively eliminating competing microbes. It is also possible that the antimicrobial peptide is a defense mechanism against potentially harmful microbes in the environment. Contig1066 encodes a protein similar to the uncharacterized protein family UPF0005 containing protein in *Brugia malayi *(GenBank accession number XP_001896958 ) and BAX inhibitor-1-like protein in wasp *Nasonia vitripennis *(GenBank accession number XP_001605379 ). BAX inhibitor-1 is a member of Bcl-2 family that suppresses programmed cell death [18]. Transmembrane BAX inhibitor motif protein (TMBI) homologs have been identified in *C. elegans*, *C. briggsae*, *C. japonica*, *C. remanei*, and *Pristioncus pacifiucs *(Wormbase). However, these genes have very low sequence similarities to *H. bacteriophora *Contig1066. BAX inhibitor-1 is involved in preventing endoplasmic reticulum stress-related programmed cell death in *Arabidopsis *[19] and humans [20].

GO assignments based on sequence similarity searches aid identification of *H. bacteriophora *distinct ESTs involved in different biological processes. Here we discuss the genes involved in some biological processes of interest in detail. A number of ESTs related to defense responses and stress responses were identified in these *H. bacteriophora *distinct ESTs (Additional File 2) based on GO assignments. Among *H. bacteriophora *distinct ESTs involved in defense response are 3 ESTs encoding a homolog of *C. elegans *SMEK (*Dictyostelium *suppressor of MEK null) homolog that is essential for DAF-16-mediated defense response to pathogenic bacteria and increased resistance to oxidative and UV-induced damage [21]. EST ES410098 encodes a heat shock protein HSP16-1 that is induced solely in response to heat shock or other environmental stresses [22]. Another 13 ESTs encoding 5 different proteins whose *C. elegans *homologs exhibit a "pathogen susceptibility increased" phenotype when silenced by RNAi [23]. However, the molecular functions of these proteins have yet to be elucidated. The defense response transcripts may be involved in the protection of entomopathogenic nematode IJs from bacterial or fungal pathogens and the insect innate immune system.

Five *H. bacteriophora *distinct ESTs involved in stress response encode a homolog to *C. elegans *catalase CTL-2 that likely is involved in protecting cells from reactive oxygen species as an antioxidant enzyme [24]. Another EST (ES742296) encodes a protein whose *C. elegans *homolog showed an "organism stress response abnormal" phenotype when silenced with RNAi [25]. Functions of other ESTs involved in stress response are yet to be clearly characterized. These transcripts related to stress responses provide workable targets for the improvement of ultraviolet, desiccation, and heat stress tolerance, traits desperately sought for improving the biological pest control potential of *H. bacteriophora*. Once the functions of these genes are determined, they can be potentially used for genetic manipulations of entomopathogenic nematodes. ESTs involved in dauer larval development, dauer entry, and dauer exit were also identified (Table 4) according to GO assignments. The infective juvenile stage of entomopathogenic nematodes is developmentally similar to the dauer stage in many bacteria feeding nematodes, including *C. elegans *and *C. briggsae*. The dauer is a developmentally arrested stage triggered by food deprivation, high population density, and other harsh environmental conditions [26]. Elucidation of this process is of specific interest in the case of entomopathogenic nematodes because the dauer juvenile is the only life stage capable of infecting insects [4].

RNA interference represents a powerful technique for analysis of gene function. An RNAi system relying on soaking in double-stranded RNA solution has been established in *H. bacteriophora *[7]. Interestingly, we were able to identify only a small number of known RNAi related genes in *H. bacteriophora *(Table 3) compared to *C. elegans *and *B. malayi *[10].

We have identified genes encoding RNAi induced silencing complex (RISC) components. One EST (EX014403) encodes a homolog of *C. elegans *TSN-1 (71% similarity at the amino acid level) and another EST (Contig211) encodes a homolog of *C. elegans *VIG-1 (45% similarity at the amino acid level). TSN-1 (Tudor staphylococcal nuclease) containing 5 staphylococcal/micrococcal nuclease domains and a tudor domain is a RISC component in *C. elegans*, *Drosophila *and mammals [27]. The purified TSN-1 from *C. elegans *was shown to have nuclease activity and therefore thought to contribute to RNA degradation in RNAi [27]. The product of the *vig-1 *gene was also shown to be a component of RISC [28]. We did not identify a member of Argonaute family in this EST set based on sequence similarity. However, we identified an EST (FF678397) encoding a protein similar to a PAZ domain containing protein from *Brugia malayi *(57% similarity at the amino acid level). Another EST (FF679415) encodes a putative homolog to *Drosophila *and human Drosha [29] rather than Dicer in *C. elegans*. These findings suggest that *H. bacteriophora *may have structurally different RNAi pathway components than its relative, *C. elegans*.

Other RNAi related genes we were able to identify are those encoding homologs of SMG-2, SMG-5, RDE-4, GFL-1, and ZFP-1. SMG-2 and SMG-5 are involved in nonsense-medicated mRNA decay (NMD) where eukaryotic mRNAs with premature stop codons are selectively and rapidly degraded [30,31]. The other three genes, *rde-4 *[32], *gfl-1 *and *zfp-1 *[33] were shown to be involved in RNAi via RNAi evidence. We currently are not able to identify a gene encoding a SID-1 homolog in *H. bacteriophora *TTO1 that was shown to be necessary for systemic RNAi [34]. However, a *sid-1 *gene has been found in *H. bacteriophora *GPS11 [3]. It is possible that more known genes may be identified when the complete genome of *H. bacteriophora *TTO1 is sequenced.

This EST project also enabled the development of genetic markers. We have identified 168 microsatellite loci from *H. bacteriophora *distinct ESTs, of which we were able to design primers for 141 based on the flanking sequences. These microsatellite markers may be useful for genetic mapping, linkage analysis, and population genetic studies. In a separate effort, microsatellite loci with 2- or 3-bp repeat units were selected for microsatellite marker development, along with the microsatellite loci enriched from genomic DNA of *H. bacteriophora *[35]. Eight polymorphic microsatellite loci were demonstrated within a Northeast Ohio population.

## Conclusion

We have generated 31,485 high quality *H. bacteriophora *ESTs representing 10,886 distinct sequences. Among these, 7,828 (71.9%) ESTs matched to proteins in GenBank's nr database. The vast majority (95.9%) of the best matches was to nematode proteins, a small portion (0.3%) to prokaryotic proteins and the remaining 3.8% to other eukaryotic proteins. GO terms were assigned to 6,685 *H. bacteriophora *distinct ESTs. "Embryonic development ending in birth or egg hatching" and "protein binding" were the most dominant terms in the categories of Biological Process and Molecular Function, respectively. This EST collection offers unprecedented opportunities for research on this unique nematode-bacterium symbiotic complex. The comparison of ESTs of *H. bacteriophora *TTO1 with those of AHPNs, FLNs, and PPNs resulted in the identification of 554 parasitic nematode-specific ESTs. These ESTs should be valuable for future research related to insect parasitism by these nematodes. We were able to identify a small number of ESTs involved in RNAi, among which is an EST encoding a Drosha homolog, suggesting structurally different RNAi pathway components from those in *C. elegans*. In addition, we have identified 157 microsatellite loci which may prove valuable once their polymorphisms are tested and validated. Overall, novel, parasitic nematode-specific, and *C. elegans *homologous genes have been identified in this EST study, greatly facilitating genome annotation, gene functional analysis, population genetic studies, and microarray development.

## Methods

### RNA isolation, cDNA library construction, and sequencing

Total RNA and poly(A) RNA were isolated from adult hermaphrodites of the isogenic line of *Heterorhabditis bacteriophora *TTO1-M31e strain propagated on a lawn of *Photorhabdus luminescens *bacterium. Poly(A) RNA was used for cDNA library construction with two different strategies. The first group of libraries were constructed using the CloneMiner™ cDNA Library Construction Kit (Invitrogen) following the manufacturer's instructions. Briefly, 2 μg single-stranded mRNA was converted into double stranded cDNA containing *att*B sequences on each end. Through site-specific recombination, *att*B-flanked cDNA was cloned into the *att*P-containing pDONR222 vector. The second group of libraries were constructed using SMART technology with modifications. Briefly, the double stranded cDNA was synthesized with SMART oligos from poly(A) RNA with SuperScript^® ^III First-Strand Synthesis System (Invitrogen) and Advantage^® ^High Fidelity 2 PCR kit (Clontech). The double-stranded cDNA was normalized with duplex-specific nuclease (Evrogen) and then was nebulized, end repaired with End Repair Kit (Lucigen), size separated, and ligated into pSMART hinc II Vector System (Lucigen). The cloning and sequencing of both pDONR222 and pSMART libraries were not directional, leading to the production of ESTs from both 5' and 3' ends. The sequences were generated by ABI 3730 machines from the cDNA libraries using and deposited in GenBank dbEST.

### Contig assembly and analysis

EST sequences in FASTA format were downloaded from GenBank dbEST. The sequences were processed by removing vector sequences with Vector NTI Advance™ 10 program. The processed EST sequences were assembled into contigs (contiguous sequences) using the ContigExpress module embedded in Vector NTI Advance™ 10 (Invitrogen). These stringent parameters of assembly (overlap length cutoff of 40 and overlap percent identity cutoff of 95%) were used to assure proper assembly. The distinct EST sequences, including the contig consensus sequences and the singleton sequences, were searched against GenBank's nr database and Wormpep190 databases in a local Linux workstation using the BLASTx algorithm [36]. The E value cutoff of the BLASTx searches was 1e-5.

### EST comparison

All nematode EST sequences were downloaded from GenBank dbEST to a local Linux workstation and formatted as a database for tBLASTx searches. Gene index (GI) numbers of all nematode EST sequences were extracted and grouped according to the categories of AHPNs, PPNs, and FLNs. The tBLASTx searches were performed in a local Linux workstation against the complete nematode EST database with the -l option enabled to restrict the database search to the list of GI's of the targeted group [7]. For example, when comparing *H. bacteriophora *ESTs to ESTs of FLNs, "-l fln.gi" was included in the command with fln.gi containing all GI numbers of EST entries from free-living nematodes. The BLAST outputs were parsed with in-house developed perl scripts to extract match information. ESTs with no significant matches to ESTs of FLNs were extracted and further searched against GenBank nr and Wormpep190 databases using BLASTx algorithm. EST entries with no significant matches to proteins of FLNs were designated parasitic nematode-specific ESTs, which were further characterized. The cutoff value of BLAST searches was 1e-5.

### Gene Ontology annotation

For assignment of Gene Ontology terms, the distinct *H. bacteriophora *ESTs were searched using the BLASTx algorithm against the annotated sequences of FASTA format in the April 2008 release of GO database. The BLAST output was parsed and terms assigned with the assistance of in-house developed perl scripts accessing the MySQL database of "mygo" in a local Linux workstation. The distribution of GO terms in each of the main ontology categories, Biological Process, Cellular Component, and Molecular Function [37], was examined. The number of *H. bacteriophora *distinct ESTs assigned in a single GO category was considered as 100% [12,38].

### Bioinformatics mining of microsatellite loci

The set of 10,886 *H. bacteriophora *distinct ESTs were searched for microsatellite loci using msatfinder v. 2.0.9 [39] in a local Linux workstation. The cut-off values of number of repeats were set to 6 for di-nucleotide loci and 5 for tri-, tetra-, penta-, and hexa-nucleotide loci. Primers were designed using Primer3 release 1.0 [40] in a local Linux workstation.

## Authors' contributions

XB conducted vector sequence removal, assembly and analysis of EST sequences and prepared the manuscript. TAC provided the poly(A) RNA for cDNA library construction. RKW, SC, and JS led groups at the Genome Center at Washington University School of Medicine in St. Louis, MO that conducted cDNA library construction, sequencing, and data deposition. PSG, BJA, TAC, SC, RG, SAH, JS, and PWS initiated the study and obtained funding. PSG supervised the study and assisted in manuscript preparation. All authors read, edited, and approved the final manuscript.

## Supplementary Material

Additional file 1 **ContigComponents**. Components of *H. bacteriophora *assembled contigs. The table includes the components of *H. bacteriophora *assembled contigs, including contig names and their comma-delimited components in GenBank accession numbers. Click here for file

Additional file 2 **defense_stress_response_genes**. *H. bacteriophora *distinct ESTs similar to *C. elegans *genes involved in defense and stress responses. This table contains *H. bacteriophora *distinct ESTs matching to *C. elegans *genes that are involved in defense and stress responses. Information of each *H. bacteriophora *distinct EST includes matching *C. elegans *gene, its description, E value of the BLASTx search, alignment, and percentage similarity. Click here for file

Additional file 3 **NematodeTaxa**. Nematode species that ESTs used in the analysis came from. The table includes the nematode species in the categories of AHPNs, FLNs, and PPNs, the clade they belong to, and their corresponding number of ESTs in GenBank when the analysis was done. Click here for file

Additional file 4 **panESTs**. Summary of BLASTx identification of parasitic nematode-specific *H. bacteriophora *ESTs. The table describes the best BLASTx matches to GenBank's nr database of the 554 parasitic nematode-specific *H. bacteriophora *ESTs, including distinct EST ID, length, and annotation of the top BLASTx hit, including accession number, length, description, E value, bit score, frame, query_start, query_end, hit_start, hit_end, positives, and identities. Click here for file

Additional file 5 **GOassignment**. Summary of GO assignment of *H. bacteriophora *distinct ESTs. The table describes the GO assignment for each of 10,886 *H. bacteriophora *distinct ESTs, of which 4,201 have "no GO assignment". The GO terms separated by semicolons include accession numbers, term descriptions, and term types. Click here for file

Additional file 6 **msat**. Summary of microsatellite loci identified in *H. bacteriophora *distinct ESTs. The table describes the microsatellite loci identified in *H. bacteriophora *distinct ESTs, including the ID and length of *H. bacteriophora *distinct EST ID, the start and stop coordinates and motif units of the microsatellite loci, GC contents of the distinct ESTs and the flanking sequences, primer sequence, length, and Tm of the left and right primers, and the size of the amplification product from these primers. The asterisk (*) marking *H. bacteriophora *distinct EST IDs denotes that the primers designed for these loci amplify other adjacent loci too. Click here for file

## References

[B1] Grewal PS, Ehlers RU, Shapiro-Ilan DI (2005). Nematodes as Biocontrol Agents.

[B2] Ciche T (2007). The biology and genome of *Heterorhabditis bacteriophora *(February 20, 2007).

[B3] Sandhu SK, Jagdale GB, Hogenhout SA, Grewal PS (2006). Comparative analysis of the expressed genome of the infective juvenile entomopathogenic nematode *Heterorhabditis bacteriophora*. Mol Biochem Parasitol.

[B4] Grewal PS, Lewis EE, Gaugler R (1997). Response of infective stage parasites (Nematoda: Steinernematidae) to volatile cues from infected hosts. J Chem Ecol.

[B5] Ciche TA, Ensign JC (2003). For the insect pathogen *Photorhabdus luminescens*, which end of a nematode is out?. Appl Environ Microbiol.

[B6] Bai X, Grewal PS, Hogenhout SA, Adams BJ, Ciche TA, Gaugler R, Sternberg PW (2007). Expressed sequence tag analysis of gene representation in insect parasitic nematode *Heterorhabditis bacteriophora*. J Parasitol.

[B7] Ciche TA, Sternberg PW (2007). Postembryonic RNAi in *Heterorhabditis bacteriophora*: a nematode insect parasite and host for insect pathogenic symbionts. BMC Dev Biol.

[B8] The C. elegans sequencing consortium (1998). Genome sequence of the nematode *C. elegans*: a platform for investigating biology. Science.

[B9] Stein LD, Bao Z, Blasiar D, Blumenthal T, Brent MR, Chen N, Chinwalla A, Clarke L, Clee C, Coghlan A (2003). The genome sequence of *Caenorhabditis briggsae*: a platform for comparative genomics. PLoS Biol.

[B10] Ghedin E, Wang S, Spiro D, Caler E, Zhao Q, Crabtree J, Allen JE, Delcher AL, Guiliano DB, Miranda-Saavedra D (2007). Draft genome of the filarial nematode parasite *Brugia malayi*. Science.

[B11] Duchaud E, Rusniok C, Frangeul L, Buchrieser C, Givaudan A, Taourit S, Bocs S, Boursaux-Eude C, Chandler M, Charles JF (2003). The genome sequence of the entomopathogenic bacterium *Photorhabdus luminescens*. Nat Biotechnol.

[B12] Quilang J, Wang S, Li P, Abernathy J, Peatman E, Wang Y, Wang L, Shi Y, Wallace R, Guo X (2007). Generation and analysis of ESTs from the eastern oyster, *Crassostrea virginica *Gmelin and identification of microsatellite and SNP markers. BMC Genomics.

[B13] Rogers A, Antoshechkin I, Bieri T, Blasiar D, Bastiani C, Canaran P, Chan J, Chen WJ, Davis P, Fernandes J (2008). WormBase 2007. Nucleic Acids Res.

[B14] Scholl EH, Thorne JL, McCarter JP, Bird DM (2003). Horizontally transferred genes in plant-parasitic nematodes: a high-throughput genomic approach. Genome Biol.

[B15] Bai C, Sen P, Hofmann K, Ma L, Goebl M, Harper JW, Elledge SJ (1996). SKP1 connects cell cycle regulators to the ubiquitin proteolysis machinery through a novel motif, the F-box. Cell.

[B16] Yan KS, Yan S, Farooq A, Han A, Zeng L, Zhou MM (2003). Structure and conserved RNA binding of the PAZ domain. Nature.

[B17] Tasiemski A, Vandenbulcke F, Mitta G, Lemoine J, Lefebvre C, Sautiere PE, Salzet M (2004). Molecular characterization of two novel antibacterial peptides inducible upon bacterial challenge in an annelid, the leech Theromyzon tessulatum. J Biol Chem.

[B18] Huckelhoven R (2004). BAX Inhibitor-1, an ancient cell death suppressor in animals and plants with prokaryotic relatives. Apoptosis.

[B19] Watanabe N, Lam E (2008). BAX inhibitor-1 modulates endoplasmic reticulum stress-mediated programmed cell death in Arabidopsis. J Biol Chem.

[B20] Lee GH, Kim HK, Chae SW, Kim DS, Ha KC, Cuddy M, Kress C, Reed JC, Kim HR, Chae HJ (2007). Bax inhibitor-1 regulates endoplasmic reticulum stress-associated reactive oxygen species and heme oxygenase-1 expression. J Biol Chem.

[B21] Wolff S, Ma H, Burch D, Maciel GA, Hunter T, Dillin A (2006). SMK-1, an essential regulator of DAF-16-mediated longevity. Cell.

[B22] Jones D, Dixon DK, Graham RW, Candido EP (1989). Differential regulation of closely related members of the hsp16 gene family in Caenorhabditis elegans. DNA.

[B23] Shapira M, Hamlin BJ, Rong J, Chen K, Ronen M, Tan MW (2006). A conserved role for a GATA transcription factor in regulating epithelial innate immune responses. Proc Natl Acad Sci USA.

[B24] Togo SH, Maebuchi M, Yokota S, Bun-Ya M, Kawahara A, Kamiryo T (2000). Immunological detection of alkaline-diaminobenzidine-negativeperoxisomes of the nematode Caenorhabditis elegans purification and unique pH optima of peroxisomal catalase. Eur J Biochem.

[B25] Cho JH, Ko KM, Singaravelu G, Ahnn J (2005). Caenorhabditis elegans PMR1, a P-type calcium ATPase, is important for calcium/manganese homeostasis and oxidative stress response. FEBS Lett.

[B26] Hu PJ (2007). Dauer (August 08, 2007).

[B27] Caudy AA, Ketting RF, Hammond SM, Denli AM, Bathoorn AM, Tops BB, Silva JM, Myers MM, Hannon GJ, Plasterk RH (2003). A micrococcal nuclease homologue in RNAi effector complexes. Nature.

[B28] Caudy AA, Myers M, Hannon GJ, Hammond SM (2002). Fragile X-related protein and VIG associate with the RNA interference machinery. Genes Dev.

[B29] Denli AM, Tops BB, Plasterk RH, Ketting RF, Hannon GJ (2004). Processing of primary microRNAs by the Microprocessor complex. Nature.

[B30] Kim JK, Gabel HW, Kamath RS, Tewari M, Pasquinelli A, Rual JF, Kennedy S, Dybbs M, Bertin N, Kaplan JM (2005). Functional genomic analysis of RNA interference in C. elegans. Science.

[B31] Anders KR, Grimson A, Anderson P (2003). SMG-5, required for C. elegans nonsense-mediated mRNA decay, associates with SMG-2 and protein phosphatase 2A. EMBO J.

[B32] Tabara H, Sarkissian M, Kelly WG, Fleenor J, Grishok A, Timmons L, Fire A, Mello CC (1999). The rde-1 gene, RNA interference, and transposon silencing in C. elegans. Cell.

[B33] Dudley NR, Labbe JC, Goldstein B (2002). Using RNA interference to identify genes required for RNA interference. Proc Natl Acad Sci USA.

[B34] Winston WM, Molodowitch C, Hunter CP (2002). Systemic RNAi in C. elegans requires the putative transmembrane protein SID-1. Science.

[B35] Bai X, Saeb ATM, Michel A, Grewal PS (2009). Isolation and characterization of microsatellite loci in the entomopathogenic nematode *Heterorhabditis bacteriophora*. Mol Ecol Resources.

[B36] Altschul SF, Madden TL, Schaffer AA, Zhang J, Zhang Z, Miller W, Lipman DJ (1997). Gapped BLAST and PSI-BLAST: a new generation of protein database search programs. Nucleic Acids Res.

[B37] Ashburner M, Ball CA, Blake JA, Botstein D, Butler H, Cherry JM, Davis AP, Dolinski K, Dwight SS, Eppig JT (2000). Gene ontology: tool for the unification of biology. The Gene Ontology Consortium. Nat Genet.

[B38] Vizcaino JA, Gonzalez FJ, Suarez MB, Redondo J, Heinrich J, Delgado-Jarana J, Hermosa R, Gutierrez S, Monte E, Llobell A (2006). Generation, annotation and analysis of ESTs from *Trichoderma harzianum *CECT 2413. BMC Genomics.

[B39] Thurston MI, Field D (2005). Msatfinder: detection and characterisation of microsatellites.

[B40] Rozen S, Skaletsky HJ, Krawetz S, Misener S (2000). Primer3 on the WWW for general users and for biologist programmers. Bioinformatics Methods and Protocols: Methods in Molecular Biology.

